# Subarachnoid Hemorrhage, Spreading Depolarizations and Impaired Neurovascular Coupling

**DOI:** 10.1155/2013/819340

**Published:** 2013-03-13

**Authors:** Masayo Koide, Inna Sukhotinsky, Cenk Ayata, George C. Wellman

**Affiliations:** ^1^Department of Pharmacology, University of Vermont College of Medicine, Burlington, VT 05405-0068, USA; ^2^Neurovascular Research Laboratory, Department of Radiology, Stroke Service and Neuroscience Intensive Care Unit, Department of Neurology, Massachusetts General Hospital and Harvard Medical School, Boston, MA 02115, USA; ^3^Gonda Multidisciplinary Brain Research Center, Bar-Ilan University, Ramat-Gan 52990, Israel

## Abstract

Aneurysmal subarachnoid hemorrhage (SAH) has devastating consequences on brain function including profound effects on communication between neurons and the vasculature leading to cerebral ischemia. Physiologically, neurovascular coupling represents a focal increase in cerebral blood flow to meet increased metabolic demand of neurons within active regions of the brain. Neurovascular coupling is an ongoing process involving coordinated activity of the neurovascular unit—neurons, astrocytes, and parenchymal arterioles. Neuronal activity can also influence cerebral blood flow on a larger scale. Spreading depolarizations (SD) are self-propagating waves of neuronal depolarization and are observed during migraine, traumatic brain injury, and stroke. Typically, SD is associated with increased cerebral blood flow. Emerging evidence indicates that SAH causes inversion of neurovascular communication on both the local and global level. In contrast to other events causing SD, SAH-induced SD decreases rather than increases cerebral blood flow. Further, at the level of the neurovascular unit, SAH causes an inversion of neurovascular coupling from vasodilation to vasoconstriction. Global ischemia can also adversely affect the neurovascular response. Here, we summarize current knowledge regarding the impact of SAH and global ischemia on neurovascular communication. A mechanistic understanding of these events should provide novel strategies to treat these neurovascular disorders.

## 1. Pathophysiology of Subarachnoid Hemorrhage

Aneurysmal subarachnoid hemorrhage (SAH) is associated with high morbidity and mortality with limited therapeutic options [1]. The major contributor to poor outcome of patients surviving the initial surge in intracranial pressure is delayed cerebral ischemia (DCI) manifesting 4–10 days after aneurysm rupture as new and otherwise unexplained neurological deficits and/or ischemic lesions within the brain [2]. Despite decades of study, mechanisms contributing to SAH-induced DCI remain controversial. For many years, a delayed and prolonged vasospasm of large conduit arteries was thought to be the major contributor to DCI and the ensuing death and disability observed in SAH patients [3, 4]. Recent data, however, challenge this view [5–7] and strongly suggest that additional mechanisms contribute to poor outcomes after SAH, including early brain injury suffered at the time of bleed [6, 8–10], blood-brain barrier disruption [11, 12], inflammation [13–15], and impaired microcirculatory function [16–19]. Evidence suggests that a pathological inversion of neurovascular coupling may play an important role in SAH pathology both in the context of spreading depolarization waves [20] and at the level of the neurovascular unit in response to focal neuronal activity [21]. 

## 2. Spreading Depression and Injury Depolarizations

Spreading depression (SD) is the historical term used to describe intense neuronal and glial depolarization events that propagate within cortical or subcortical grey matter at a rate of 2–4 mm/min regardless of functional divisions or arterial boundaries [22]. Initially implicated in migraine aura, SD-like depolarization waves also occur in stroke and traumatic brain injury [23, 24]. The pivotal event during SD is a massive K^+^ efflux that increases extracellular K^+^ concentration to >40 mM. Massive influx of Ca^2+^, Na^+^, and water accompanies the K^+^ efflux and triggers uncontrolled release of neurotransmitters, most importantly the excitatory amino acid glutamate. Released K^+^ and glutamate are believed to depolarize other neurons in the vicinity, and SD slowly propagates in grey matter by way of contiguity. Therefore, extracellular medium, including the perivascular space, is flooded with K^+^ and neurotransmitters that are vasoactive. Because complete membrane depolarization precludes action potentials and synaptic transmission, SD is associated with suppression of all spontaneous or evoked electrical activity. Consequently, the normal neuronal influence on the vasculature is absent at least until the ability of neurons to generate action potentials returns, which can take several minutes. Moreover, there is ample evidence suggesting that physiological neurovascular coupling is impaired not only during the depolarization but for hours after the SD event [25–28]. 

SD is triggered when a minimum critical volume of brain tissue is simultaneously depolarized. Therefore, cerebral ischemia, anoxia, and other forms of brain injury can all trigger SD. Both animal models and clinical studies have clearly demonstrated the occurrence of SD waves associated with traumatic brain injury, cerebral ischemia, and subarachnoid hemorrhage [24, 29–33]. With respect to the emergence of SD after SAH, a number of potentially interacting factors have been implicated. These SD promoting factors and influences from subarachnoid blood include increased extracellular K^+^ combined with decreased nitric oxide bioavailability [30, 34, 35], oxyhemoglobin [34, 36, 37], and endothelin-1 [37–40]. Such spreading injury depolarizations occur repetitively over hours and days and propagate throughout the unhealthy, but not yet depolarized or necrotic tissue (e.g., ischemic penumbra). Indeed, such injury depolarizations are indistinguishable from SD when they often propagate into noninjured tissue. The existence of injury depolarizations has been recognized for decades, and their detrimental effect on tissue outcome has been attributed to their profound metabolic impact. More recently, however, an additional mechanism was discovered that exacerbates the energy supply-demand mismatch in injured brain. This novel mechanism, termed inverse or vasoconstrictive neurovascular coupling, leads to a reduction in tissue perfusion instead of the usual hyperemia SD causes in normal brain tissue.

## 3. Influence of Spreading Depolarizations on Cerebral Blood Flow

In most species and studies, and under normal physiological conditions, SD is typically associated with a profound hyperemic response that starts shortly after the onset of depolarization and outlasts it by a few minutes [41–46]. As SD has a profound metabolic impact on brain tissue [47], this increase in the flow of nutrients enables neurons to recover from the massive ion and water imbalance occurring during SD events. However, the vasomotor impact of SD can also be complex. For example, a brief hypoperfusion occasionally precedes the hyperemia, its onset coinciding with the onset of depolarization. This initial hypoperfusion is augmented by nitric oxide (NO) inhibition, particularly when extracellular potassium ([K^+^]_e_) is artificially elevated [34, 48–51]. In mice, the initial vasoconstriction is much more pronounced and hyperemia is completely absent [41]. Vascular response also appears to vary depending on vessel caliber and/or cortical depth. Larger pial surface arterioles respond to SD with a small initial constriction followed by dilation, whereas smaller parenchymal arterioles mainly constrict [52]. In general, vasoconstrictive tone develops during the depolarization, followed by a vasodilator tone during repolarization, and then a second vasoconstrictive phase that can last up to an hour [53]. The magnitude and time course of these opposing vasomotor components vary depending on species and experimental conditions, can be modulated physiologically and pharmacologically, and determine the final morphology of the hemodynamic response [53]. Altogether, these observations suggest that SD exerts multiple opposing vasomotor effects on blood vessels, with vasodilation predominating in healthy tissue.

Pathological circumstances such as ischemic stroke or subarachnoid hemorrhage modulate the magnitude and timing of the vasomotor components. Under such conditions, the vascular response becomes predominantly vasoconstrictive, that is, inverted [46, 54, 55]. This likely represents a shift in the balance of vasomotor influences to vasoconstriction. As a result, injury depolarizations cause hypoperfusion rather than hyperemia that could potentially lead to a downward spiral of increased brain injury [33, 56, 57]. In ischemic penumbra, the more ischemic the tissue is (i.e., closer to the core), the more severe the vasoconstrictive component becomes [33, 56, 58–60]. Such conditions can be recreated to transform the CBF response. For example, in the presence of extravascular hemoglobin and elevated [K^+^]_e_ or low glucose, mimicking subarachnoid hemorrhage, SD is associated with severe vasoconstriction [34]. Induced hypoxia and hypotension independently augment the hypoperfusion component of the hemodynamic response to SD and significantly diminish the hyperemia [61]. Although hypotension appears to be more potent than hypoxia in this regard, combined hypoxia and hypotension, most closely mimicking ischemic penumbra, transforms the predominantly dilator response into a biphasic one. Neither induced hyperoxia nor hyperglycemia restores the CBF response [55, 62], suggesting that cerebral perfusion pressure affects SD-mediated vascular responses by a mechanism unrelated to tissue energy status. 

Despite the fact that SD in normal cortex is not damaging, this severe vasoconstrictive response can lead to injury and cell death, even in the absence of any preexisting energy depletion [36]. Indeed, injury depolarizations worsen tissue and neurological outcome in focal cerebral ischemia and other brain injury states including aneurysmal SAH [20, 29, 31, 57, 63]. Conversely, drugs that are known to inhibit cortical spreading depression, such as NMDA receptor antagonists MK-801, diminish the severity of episodic hypoperfusions and prevent the expansion of severely hypoperfused cortex, eventually reducing the infarct size [20, 29, 31, 33, 63]. However, in vivo studies have shown the efficacy of MK-801 to prevent SD was greatly diminished when extracellular K^+^ was elevated [64]. Topical application of vasodilator agents such as nitric oxide and the L-type voltage-dependent Ca^2+^ channel blocker nimodipine reverts the vasoconstrictive response to vasodilation [34, 54, 65]. Therefore, mechanisms transforming the CBF response from hyperemia to hypoperfusion during injury depolarizations may be targeted to interrupt the vicious cycle and improve tissue outcome. Further, recent evidence suggests SAH can have a profound impact on the individual neurovascular unit leading to inversion of neurovascular coupling in the absence of SD.

## 4. Functional Hyperemia at the Level of the Neurovascular Unit

Functional hyperemia and neurovascular coupling are terms often used interchangeably to describe increased cerebral blood flow (CBF) in brain regions with enhanced neuronal activity, which forms the basis of functional magnetic resonance imaging (fMRI) [66]. This localized vasodilation to meet activity-dependent metabolic demand involves interplay of cells comprising the neurovascular unit—neurons, astrocytes and intracerebral (parenchymal) arterioles [67–69]. Astrocytes act as key intermediaries in the neurovascular response, structurally having close “synapse-like” associations with neurons as well as processes (astrocytic endfeet) that completely encase parenchymal arterioles. Over the past decade, numerous investigators primarily using cortical brain slices have provided evidence linking increased neuronal activity and nerve-mediated glutamate release to the activation of astrocytic metabotropic glutamate receptors (mGluRs), inositol triphosphate-(IP_3_-) mediated increase in astrocyte Ca^2+^ and Ca^2+^-dependent release of vasodilator influences from astrocytic endfeet [68, 70–75]. Excitatory and inhibitory interneurons may also modulate the neurovascular coupling process via an influence on astrocyte Ca^2+^ or through direct effects on parenchymal arterioles [76–78]. Multiple vasodilator mechanisms have been proposed to contribute neurovascular coupling. Elevations in astrocytic endfoot Ca^2+^ have been linked to increased Ca^2+^-dependent phospholipase A_2_ (PLA_2_) activity and release of vasodilatory arachidonic acid metabolites. These include prostaglandin E_2_ (PGE_2_) produced by cyclooxygenase-1, and epoxyeicosatrienoic acids (EETs) produced by the cytochrome P450 epoxygenase, CYP 2C11 [70, 71, 79–81]. In addition, large conductance Ca^2+^-activated K^+^ (BK) channels are localized to astrocytic endfeet [82] and play a key role in neurovascular coupling [69, 83, 84]. Endfoot BK channel activation by moderate increases in astrocytic Ca^2+^ causes localized increases in K^+^ in the perivascular space that stimulate inwardly rectifying K^+^ (K_ir_) channels located on the smooth muscle of parenchymal arterioles leading to membrane potential hyperpolarization and vasodilation [69, 71, 75, 83–85]. In sum, increased endfoot Ca^2+^ is a critical step linking local neuronal activity to parenchymal arteriolar dilation. 

## 5. Neurovascular Coupling Can Also Lead to Pathological Vasoconstriction

In vitro studies have reported that under certain conditions, neuronal activation can also lead to parenchymal arteriolar constriction [84, 86–88]. Neurally evoked vasoconstriction likely represents a pathological phenomenon promoting a decrease, rather than an increase in blood flow to metabolically active brain tissue. Mulligan and MacVicar [88] were the first to report this phenomenon in brain slices using the neurotransmitter norepinephrine or the release of caged Ca^2+^ to increase Ca^2+^ levels in the astrocyte soma. These constrictions were abolished by blockers of Ca^2+^-sensitive PLA_2_ activity and the CYP4a-mediated metabolism of arachidonic acid to the vasoconstrictor 20-hydroxyeicosatetraenoic acid (20-HETE). Both neurally mediated vasodilation and vasoconstriction have been observed in the retina [87]. In the retina, the balance between constriction and dilation was dependent upon nitric oxide (NO) levels, with 20-HETE synthesis contributing to constriction. Work by Girouard et al. [84] demonstrated that the level of astrocytic endfoot Ca^2+^ and endfoot BK channel activity dictate the polarity of the diameter changes caused by neuronal stimulation in cortical brain slices. These investigators observed that modest increases in endfoot Ca^2+^ (<500 nM) and endfoot BK channel activity lead to enhanced arteriolar K_ir_ activity, membrane potential hyperpolarization, and vasodilation. However, more robust elevations in endfoot Ca^2+^ (>500 nM) lead to sufficient BK channel-mediated K^+^ efflux from endfeet causing arteriolar smooth muscle membrane potential depolarization and constriction. Further, modest elevation of bulk extracellular K^+^ also caused inversion of neurovascular coupling from vasodilation to vasoconstriction. Thus, several factors including astrocyte endfoot Ca^2+^ levels, extracellular K^+^ concentration and endfoot BK channel activity can influence the polarity and amplitude of the neurovascular response.

## 6. Inversion of Neurovascular Coupling from Vasodilation to Vasoconstriction after Subarachnoid Hemorrhage

**Figure 1:**
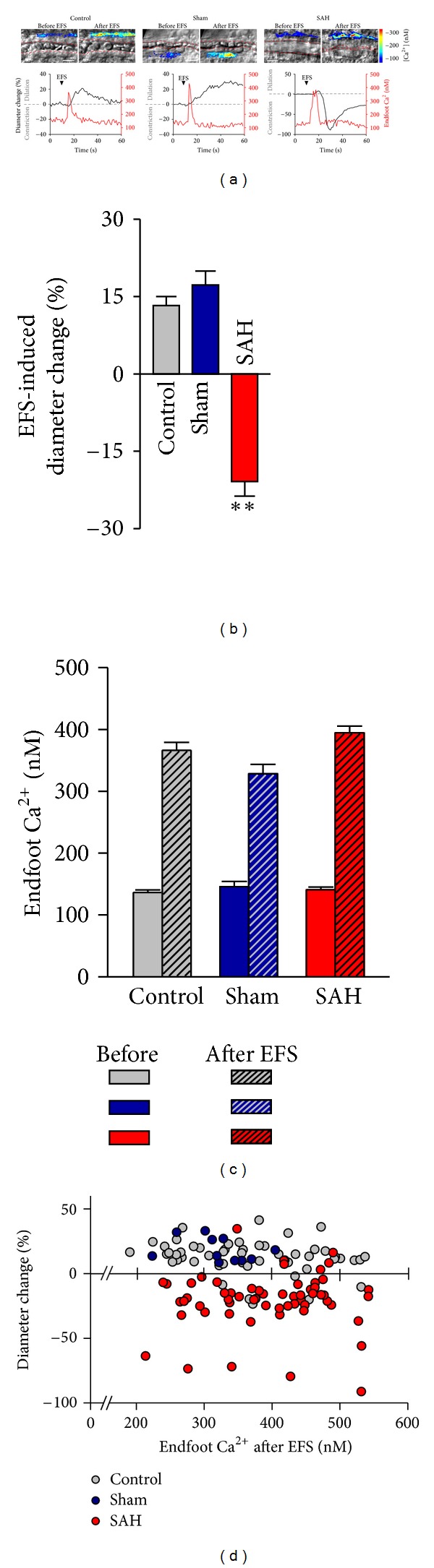
Inversion of neurovascular coupling in cortical brain slices from SAH model animals. (a) (Upper) Infrared-differential interference contrast images from brain slices of control, sham-operated, and SAH model rats before/after electrical field stimulation (EFS). Parenchymal arterioles were preconstricted with U46619 (100 nM). Dashed lines in red display the intraluminal diameter of parenchymal arterioles. Overlapping pseudocolor-mapped Ca^2+^ levels in astrocyte endfeet were obtained by simultaneous imaging using the fluorescent Ca^2+^ indicator fluo-4 and two-photon microscopy. Scale bar: 10 *μ*m. (Lower) Simultaneous recordings of EFS-induced changes in diameter and estimated endfoot Ca^2+^ concentrations obtained from brain slices depicted in upper images. (b)–(d) Summary data of EFS-evoked changes in arteriolar diameter and astrocytic endfoot Ca^2+^ obtained from control (*n* = 53), sham-operated (*n* = 11), and SAH model (*n* = 59) animals. Diameter changes were expressed as percentage of the diameter in the same point before EFS as 100%. **P < 0.01 by one-way ANOVA followed by host hoc comparison of means using the Tukey test (modified from Koide et al. [21]).

To examine the impact of experimental SAH on neurovascular coupling, our laboratory has used a combination of multiphoton confocal imaging and infrared-differential interference contrast (IR-DIC) microscopy to simultaneously measure astrocytic endfoot Ca^2+^ and parenchymal arteriolar diameter in cortical brain slices from SAH model rats [21]. Neurovascular responses were evoked using electrical field stimulation (EFS) of neurons using parameters that did not directly affect astrocytes or parenchymal arterioles. In brain slices from control and sham-operated animals, neuronal activation caused the anticipated increase in astrocytic endfoot Ca^2+^ and vasodilation. This vasodilation was greatly diminished by paxilline, a BK channel blocker, consistent with involvement of endfoot BK channels [69, 83, 84]. In marked contrast, a similar level of neuronal activation and elevation in endfoot Ca^2+^ caused vasoconstriction rather than vasodilation in brain slices from SAH model animals (Figure 1). This SAH-induced shift in neurovascular coupling from vasodilation to vasoconstriction likely represents a pathological response that could locally limit blood flow to cortical regions and was not due to increased 20-HETE or prostaglandin production. However, neurally evoked vasoconstriction after SAH was abolished by block of endfoot BK channels. Our evidence suggests the inversion of neurovascular coupling after SAH is due to increased basal endfoot BK channel activity and increased K^+^ in the restricted perivascular space between astrocytic endfeet and parenchymal arteriolar smooth muscle. This abnormal elevation of basal perivascular K^+^ combined with “normal” BK channel-mediated K^+^ efflux stimulated by neuronal activity elevates K^+^ above the dilation/constriction threshold, switching the polarity of arteriolar responses to vasoconstriction. Consistent with this interpretation, increasing concentrations of extracellular K^+^ elicit a bimodal response in isolated parenchymal arterioles [21, 83, 84]. Modest increases in K^+^ (<20 mM) induce smooth muscle hyperpolarization and arteriolar dilation through activation of K_ir_ channels expressed on arteriolar myocytes [89]. However, K^+^ increases greater than ~20 mM cause a depolarizing shift in the K^+^ equilibrium potential (E_K_) sufficient to increase the activity of voltage-dependent Ca^2+^ channels leading to enhanced Ca^2+^ influx and vasoconstriction. Although the vascular responses are inverted after SAH, both neurovascular responses (i.e., vasodilation in control animals and vasoconstriction in SAH animals) involve the same mechanistic elements: elevated astrocytic endfoot Ca^2+^ and K^+^ efflux mediated by endfoot BK channels with the polarity of the vascular response dictated by basal perivascular K^+^ levels.

**Figure 2:**
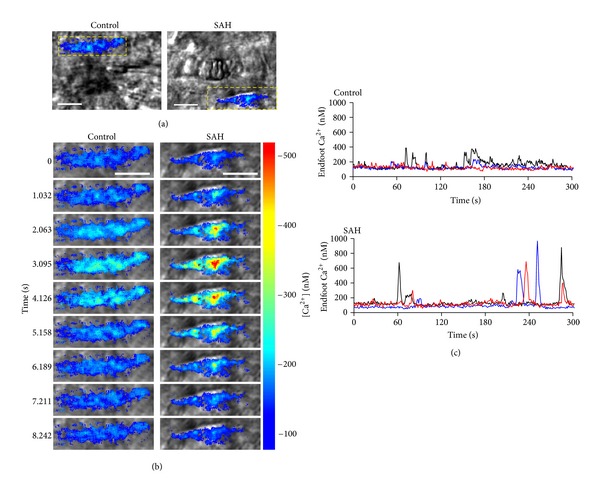
Increased amplitude of spontaneous Ca^2+^ oscillations in astrocyte endfeet following SAH. (a)-(b) Representative images of spontaneous Ca^2+^ oscillation in astrocyte endfeet in brain slices from control and SAH model animals. (b) Time laps images from the area within the yellow dotted box in Figure 2(a). Scale bar: 10 *μ*m. (c) Spontaneous Ca^2+^ oscillations in a brain slice from control (upper) and SAH model (lower) animals. Traces were obtained from 1.2 × 1.2-*μ*m regions of interest placed on distinct astrocyte endfeet in 5 min recordings without stimulation (modified from Koide et al. [21]).

**Figure 3:**
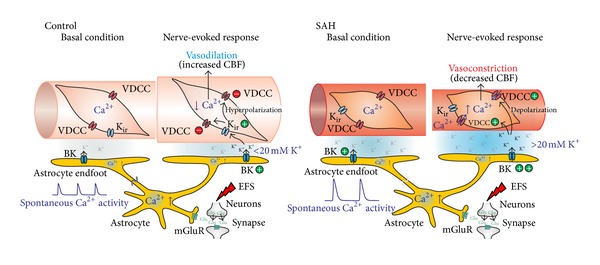
Schematic model liking SAH to inversion of neurovascular coupling. In control animals, EFS causes elevated cytoplasmic Ca^2+^ in astrocytes leading to increased BK channel activity and modest (<20 mM) increases in perivascular K^+^, promoting vasodilation. SAH increases the magnitude of spontaneous astrocytic Ca^2+^ oscillations and basal activity of BK channels, elevating K^+^ in restricted perivascular space. The summation of increased basal perivascular K^+^ and “normal” nerve-evoked astrocyte BK channel activity results in extracellular K^+^ concentrations that exceed the dilation-constriction threshold (~20 mM), inducing vasoconstriction. BK: large conductance Ca^2+^-activated K^+^ channel, CBF: cerebral blood flow, EFS: electrical field stimulation, Glu: glutamate, K_ir_: inward rectifier K^+^ channel, mGluR: metabotropic glutamate receptor, VDCC: voltage-dependent Ca^2+^ channel (modified from Koide et al. [21]).

Our data also indicate fundamental changes in the resting activity of astrocyte Ca^2+^ signaling underlying SAH-induced elevation in basal perivascular [K^+^], leading to inversion of neurovascular coupling. In addition to responding to neurally released signals, astrocytes exhibit spontaneous Ca^2+^ oscillations [90]. These Ca^2+^ oscillations occur in both soma and endfeet and have been observed in isolated brain slices [90, 91] and in vivo [92–94]. This spontaneous activity occurs in the presence of Na^+^ channel blocker tetrodotoxin to inhibit neuronal action potentials and represent intracellular Ca^2+^ release events from the endoplasmic reticulum [91]. An increase in the frequency of spontaneous astrocytic Ca^2+^ events in mouse models of Alzheimer's disease has been linked to vascular instability in vivo [94]. In brain slices from SAH model animals, we observed a marked increase in the amplitude of these events [21] (Figure 2). After SAH, the mean peak amplitude of spontaneous Ca^2+^ oscillations in astrocyte endfeet was ~490 nM compared to a mean peak amplitude ~320 nM in brain slices from control animals. In comparison, neurally-evoked increases in astrocytic Ca^2+^ were ~350 nM in both control and SAH animals. Considering that EFS-induced increases in astrocytic endfeet Ca^2+^ have been shown to induce K^+^ efflux through endfoot BK channels, spontaneous Ca^2+^ events are also likely capable of activating endfoot BK channels. Based on these observations, it is conceivable that higher amplitude spontaneous Ca^2+^ events following SAH enhance BK channel activity contributing to increased basal K^+^ in restricted perivascular space (Figure 3). Factors leading to higher amplitude spontaneous Ca^2+^ events after SAH are not currently known; however, determining their identity will provide valuable new information in the search for finding new therapeutic strategies to help SAH patients. 

## 7. Impact of Global Ischemia on Neurovascular Coupling

Global cerebral ischemia represents a generalized reduction in brain blood flow caused by, for example, cardiac arrest, shock, asphyxia, and strokes including SAH. The impact of global ischemia on brain function can range from relatively mild and temporary cognitive impairment to brain death, depending on the severity and length of the ischemic insult. Multiple mechanisms have been implicated in neuronal injury caused by global cerebral ischemia including neurotransmitter (e.g., glutamate) toxicity, cortical spreading depression, inflammation, and apoptosis [95]. Emerging evidence indicates that global ischemia may also influence neurovascular coupling. In rats, moderate, temporary forebrain ischemia can be achieved by a combination of bilateral carotid artery occlusion and controlled hypotension via the withdrawal of blood. Using this approach, Zhou et al. [96] examined the impact of 15 minutes of ischemia and reperfusion on the ability of whisker stimulation to increase relative cerebral blood flow (rCBF) to the somatosensory cortex using laser speckle imaging. Prior to the ischemic insult, rCBF to the somatosensory cortex increased ~10% in response to whisker stimulation. Following ischemia and 20 minutes of reperfusion, increased rCBF to whisker stimulation was slightly diminished and response time increased; responses returned to preischemic levels within two hours. Recently, Baker et al. examined varying levels of global forebrain ischemia on the ability of forepaw stimulation to increase cerebral blood flow in the somatosensory cortex of rats [97]. Neurovascular coupling was attenuated with increasing levels of ischemia, with severe ischemia (60% reduction in global cerebral blood flow) causing greater than a 90% reduction in the neurovascular response. The attenuation of neurovascular coupling associated with severe global ischemia lasts for several days following reperfusion [98]. Currently, little information is available regarding the cellular mechanisms contributing to decreased neurovascular coupling associated with global ischemia. However, it is likely that ischemia may impact more than one component of the neurovascular unit. For example, ischemia has been shown to impair cerebral artery function that may limit vasodilation [99, 100]. Further, global ischemia has been shown to alter expression of K^+^-selective ion channels and TRPV4 nonselective cation channels in astrocytes from rat hippocampus [101, 102]. 

Global cerebral ischemia may also contribute to brain pathologies associated with SAH. Immediately following cerebral aneurysm rupture, increased intracranial pressure caused by the release of blood into the subarachnoid space can lead to transient global ischemia and contribute to a cascade of events referred to as “early brain injury” [6, 10]. Further, delayed blood-induced vasospasm of brain surface conduit arteries [103] and enhanced constriction of resistance-size cerebral arteries and arterioles [18, 104, 105] may also reduce blood flow to ischemic levels, contributing to the development of delayed ischemic neuronal deficits. Data presented above indicate that both SAH and global ischemia can lead to decreased neurovascular coupling. However, a marked difference regarding the influence of SAH and global ischemia on neurovascular coupling is apparent; SAH causes inversion of the neurovascular response from vasodilation to vasoconstriction whereas global ischemia causes a decrease in the magnitude of the dilation to evoked neuronal activity.

## 8. Conclusions

Subarachnoid hemorrhage is a multifaceted pathology exhibiting both acute and long-term injury to the brain. It is now clear that SAH profoundly impacts neuronal influences on the vasculature leading to decreased cerebral blood flow that can exacerbate the extent of brain damage. One type of SAH-induced impaired neurovascular signaling arises in the context of SD that can impact large areas of cortical and subcortical grey matter. In the absence of SAH, SD is most frequently associated with a hyperemic response, that is, an increase in cerebral blood flow. However, SAH causes an inversion of the SD-induced neurovascular response leading to vasoconstriction and decreased blood flow to tissue during a time of high metabolic demand. Recently, it has also been shown that SAH can cause inversion of neurovascular coupling at the level of the individual neurovascular unit. Physiologically, coordinated activity of neurons, astrocytes, and parenchymal arterioles ensures increase local blood flow to active neurons in specific regions of the brain engaged in task-dependent processes. After SAH the neurovascular response to neuronal activation switches from vasodilation to vasoconstriction; this also promotes a pathological decrease in the flow of oxygen and nutrients to metabolically active neurons. Evidence suggests that elevated perivascular K^+^ due to the enhanced amplitude of spontaneous Ca^2+^ signaling events in astrocytic endfeet may underlie this inversion of neurovascular coupling, consistent with a bimodal effect of extracellular K^+^ to cause vasodilation at concentrations below 20 mM and constriction when this threshold of 20 mM is exceeded. Presently mechanisms associated with inversion of the neurovascular response caused by SAH-induced SD have not completely been resolved. However, inversion of SD-induced neurovascular response likely reflects a combination of increased extracellular K^+^ and the impact of SAH on the relative balance of vasoconstrictor and vasodilator influences. Development of agents and approaches to prevent SAH-induced inversion of neurovascular coupling may provide a much needed additional therapeutic option for SAH patients.
